# Associations of probiotics combined with Tongli Gongxia Chinese medicine with intestinal barrier biomarkers and day 7 short-chain fatty acids in patients with severe acute pancreatitis

**DOI:** 10.3389/fcimb.2026.1837621

**Published:** 2026-06-29

**Authors:** Xiong Xiao, Shu Li, Liyuan Guo, Yan Wang

**Affiliations:** 1 Department of Traditional Chinese Medicine, Chengdu Fifth People’s Hospital/The Affiliated Fifth People’s Hospital of Chengdu University of Traditional Chinese Medicine, Chengdu, China; 2 Department of Traditional Chinese Medicine, The Chengdu Wenjiang People’s Hospital, Chengdu, China

**Keywords:** intestinal barrier, probiotics, severe acute pancreatitis, short-chain fatty acid, traditional Chinese medicine

## Abstract

**Background:**

Severe acute pancreatitis (SAP) is characterized by intestinal barrier dysfunction and altered short-chain fatty acid (SCFA) profiles, contributing to bacterial translocation and systemic complications. This study examined whether receipt of probiotics combined with Tongli Gongxia Chinese medicine was associated with intestinal barrier biomarker changes and day 7 fecal SCFA concentrations in SAP patients.

**Methods:**

This single-center retrospective cohort study included 240 SAP patients hospitalized between January 2019 and December 2024. After 1:1 propensity score matching, 200 patients (100 per group) were analyzed. The combination therapy group received multi-strain probiotics and Tongli Gongxia formula (Dachengqi Decoction) initiated within 72 hours of admission, plus standard therapy. The control group was restricted to patients receiving standard therapy alone during the corresponding early-treatment window. Primary outcomes were changes in four serum intestinal barrier biomarkers (diamine oxidase [DAO], D-lactate, lipopolysaccharide [LPS], and intestinal fatty acid-binding protein [I-FABP]) from baseline to day 7. Secondary outcomes included day 7 fecal SCFA concentrations, clinical outcomes, and safety events. Because baseline fecal SCFA samples were not available, SCFA analyses were interpreted as cross-sectional day 7 comparisons rather than treatment-induced changes.

**Results:**

Combination therapy was associated with greater reductions in barrier dysfunction biomarkers: ΔDAO -2.6 U/L (95% CI -3.8 to -1.4, p<0.001), ΔD-lactate -1.3 mmol/L (95% CI -1.9 to -0.7, p<0.001), ΔLPS -0.09 EU/mL (95% CI -0.13 to -0.05, p<0.001), and ΔI-FABP -216 pg/mL (95% CI -328 to -104, p<0.001). Day 7 SCFA concentrations were higher in the combination therapy group: total SCFAs 105.6 vs 85.0 μmol/g (p=0.001), including butyrate 14.6 vs 10.8 μmol/g (p=0.003). ICU stay was shorter by 2 days (11 vs 13 days, p=0.03). No probiotic-associated infections occurred. Diarrhea rates were similar (28% vs 18%, p=0.09). Conclusions: In this retrospective matched cohort, probiotics combined with Tongli Gongxia Chinese medicine were associated with greater improvement in intestinal barrier biomarkers, higher day 7 fecal SCFA concentrations, shorter ICU stay, and acceptable safety. These hypothesis-generating findings merit investigation in randomized trials.

## 1 Introduction

Severe acute pancreatitis (SAP) is a critical condition characterized by systemic inflammatory response syndrome and multiple organ dysfunction, with mortality rates ranging from 15% to 30% despite advances in intensive care management (Mederos et al., 2021; Iannuzzi et al., 2022). A hallmark pathophysiological feature of SAP is the disruption of intestinal barrier integrity, which facilitates bacterial translocation, endotoxin release, and the development of secondary infections that significantly worsen clinical outcomes (Mao et al., 2009; Capurso et al., 2019). The intestinal barrier comprises physical, chemical, immunological, and microbial components that work synergistically to maintain homeostasis, and its dysfunction in SAP has been increasingly recognized as a therapeutic target (Guo et al., 2014).

Short-chain fatty acids (SCFAs), particularly acetate, propionate, and butyrate, are the principal metabolic products of bacterial fermentation of dietary fibers and resistant starches in the colon (Koh et al., 2016). These molecules serve multiple critical functions including providing energy substrates for colonocytes, strengthening tight junction proteins, modulating immune responses, and maintaining epithelial barrier integrity (Liu et al., 2018; Parada Venegas et al., 2019). In the context of SAP, the convergence of factors such as fasting, stress-induced dysbiosis, and broad-spectrum antibiotic administration typically leads to profound reductions in SCFA-producing bacteria and consequently diminished SCFA concentrations (Gerritsen et al., 2011; Tan et al., 2015). This metabolic deficiency exacerbates barrier dysfunction and perpetuates the cycle of inflammation and organ failure.

Probiotics have emerged as a potential therapeutic intervention to modulate microbial balance and support intestinal barrier function through competitive colonization, pathogen exclusion, and stimulation of epithelial repair mechanisms (Gou et al., 2014; Kothari et al., 2019). However, their use in SAP remains controversial, with some studies suggesting benefits while others have raised safety concerns regarding potential probiotic-associated sepsis in severely immunocompromised patients (Besselink et al., 2008; Barraud et al., 2013). These conflicting findings underscore the need for careful patient selection, rigorous safety monitoring, and cautious interpretation of observational findings.

Traditional Chinese medicine (TCM) has been utilized for centuries in the management of acute abdominal conditions. The therapeutic principle of “Tongli Gongxia” (promoting passage and attacking downward) represents a classical approach to treating conditions characterized by internal heat accumulation and stagnation, typically manifested as abdominal distension, constipation, and systemic toxicity (Chen et al., 2020). The representative formula, Dachengqi Decoction, comprises herbs such as Rheum palmatum (rhubarb), Magnolia officinalis (magnolia bark), Citrus aurantium (bitter orange), and Natrii Sulfas (mirabilite), which are believed to promote intestinal motility, facilitate the elimination of metabolic waste products, and modulate the intestinal microenvironment (Zhang et al., 2015; Hu et al., 2018). Modern pharmacological investigations have revealed that these herbs contain bioactive compounds that influence gut motility, alter luminal pH, and potentially create favorable conditions for beneficial microbial growth (Gao et al., 2021; Dai et al., 2023).

The rationale for combining probiotics with Tongli Gongxia Chinese medicine in SAP stems from potentially complementary actions on intestinal transit, luminal ecology, and epithelial barrier homeostasis. While probiotics may introduce beneficial microorganisms or metabolites, Tongli Gongxia herbs may enhance intestinal transit, reduce pathogenic bacterial overgrowth in the small intestine, and create a luminal environment that could be compatible with SCFA production by modulating substrate availability and fermentation conditions (Yang et al., 2021; Yang et al., 2022). Because the present study did not include separate probiotic-only and Tongli-only comparison groups, it was not designed to isolate synergistic effects, and the combined exposure should be interpreted as a real-world integrative treatment strategy rather than proof of pharmacological synergy.

Despite the theoretical appeal of this combination strategy, existing literature predominantly consists of small-scale studies with significant methodological heterogeneity, and few investigations have systematically assessed both intestinal barrier biomarkers and comprehensive SCFA profiles as integrated endpoints. Furthermore, the temporal dynamics of barrier recovery and the relationship between day 7 metabolic profiles and clinical outcomes remain incompletely characterized in real-world clinical settings.

Therefore, the present study employed a single-center retrospective cohort design with propensity score matching and multivariable adjustment to evaluate associations between early receipt of probiotics combined with Tongli Gongxia Chinese medicine and intestinal barrier biomarker trajectories, day 7 SCFA concentrations, and clinical outcomes in SAP patients. We hypothesized that the combination exposure would be associated with greater reductions in barrier dysfunction biomarkers and higher day 7 SCFA concentrations compared with standard therapy alone, while recognizing that causal effects and synergy cannot be established from this observational design. The findings may inform the design of future prospective randomized controlled trials and contribute to evidence-based integration of complementary therapeutic approaches in critical care management of SAP.

## 2 Methods

### 2.1 Study design and ethics

#### 2.1.1 Study type and setting

This study was designed as a single-center retrospective observational cohort study, with study subjects derived from consecutive SAP patients hospitalized at Chengdu Fifth People’s Hospital between January 1, 2019, and December 31, 2024. The investigation utilized electronic medical records, laboratory information systems, and medication administration records as primary data sources. Importantly, the retrospective nature of this study ensured that no alterations were made to established clinical pathways or treatment protocols during the data collection period. All therapeutic decisions, including the administration of probiotics and Tongli Gongxia Chinese medicine, were made by attending physicians based on clinical judgment and institutional protocols rather than predetermined research protocols.

#### 2.1.2 Data protection

The study was reviewed and approved by the Ethics Committee of Chengdu Fifth People’s Hospital. Given the retrospective nature of the study and the minimal risk posed by secondary analysis of existing de-identified clinical data, the ethics committee granted a waiver of informed consent requirement. The study was conducted in accordance with the ethical principles of the Declaration of Helsinki and institutional regulations governing retrospective human-subject research.

All patient data underwent rigorous de-identification procedures prior to analysis. Personal identifiers including names, medical record numbers, precise dates of birth, and other potentially identifying information were removed or encrypted using irreversible hashing algorithms. The de-identified dataset was stored on secure, password-protected institutional servers with restricted access limited to authorized research personnel. All data access and manipulation activities were logged in audit trails to ensure accountability and traceability. The analysis was conducted within the hospital’s controlled computing environment that complies with relevant data protection regulations and institutional information security policies. No patient data were transferred to external locations or cloud-based storage systems. The research team adhered to strict confidentiality protocols throughout the study duration, and all study personnel completed institutional training in human subjects research ethics and data protection prior to study initiation.

### 2.2 Study population: inclusion and exclusion criteria and group assignment

#### 2.2.1 Inclusion criteria

Patients were considered eligible for inclusion in the study if they met all of the following criteria: (1) Age ≥18 years at the time of hospital admission, ensuring that only adult patients were included in the analysis. (2) First-time admission for acute pancreatitis that met established criteria for SAP classification according to the revised Atlanta classification, which defines SAP as acute pancreatitis with persistent organ failure (>48 hours) involving one or more organ systems. The diagnosis of acute pancreatitis required at least two of three features: characteristic abdominal pain, serum lipase or amylase at least three times the upper limit of normal, and characteristic findings on contrast-enhanced computed tomography or magnetic resonance imaging. (3) Completion of baseline measurements for at least two of the four intestinal barrier biomarkers (DAO, D-lactate, LPS, I-FABP) within 72 hours of admission, ensuring adequate baseline characterization of barrier function. (4) At least one follow-up measurement of barrier biomarkers obtained on day 3 or day 7 of hospitalization, allowing for assessment of temporal changes. (5) Availability of fecal samples for SCFA analysis on day 7, or availability of stored samples that could be retrospectively analyzed for SCFA content, ensuring completeness of metabolic assessment.

#### 2.2.2 Exclusion criteria

Patients were excluded from the study if they met any of the following criteria: (1) Pre-existing inflammatory bowel disease (including Crohn’s disease and ulcerative colitis), short bowel syndrome, or active gastrointestinal malignancy, as these conditions could independently affect intestinal barrier function and SCFA metabolism and confound the interpretation of treatment effects. (2) Systematic use of probiotics or Tongli Gongxia-type Chinese herbal formulations within one month prior to admission, to avoid carryover effects from prior treatments that might obscure the impact of in-hospital interventions. (3) Concurrent participation in interventional clinical trials targeting intestinal metabolism or microbiome modulation, which could introduce competing interventions that confound outcome assessment. (4) Death or transfer to another institution within 24 hours of admission, as such patients would have insufficient exposure to hospital-based interventions and inadequate follow-up data. (5) Critical missing data for key variables that precluded reliable multiple imputation, specifically defined as missing data for more than three of the four primary barrier biomarkers at baseline or complete absence of any follow-up measurements.

#### 2.2.3 Group assignment and exposure definition

Patients were assigned to the combination therapy group if they received both multi-strain probiotics and Tongli Gongxia Chinese medicine initiated within 72 hours of admission and continued for at least 3 consecutive days, in addition to standard therapy. The 72-hour window was selected based on clinical considerations that early intervention during the initial inflammatory surge might be most relevant to barrier deterioration. Patients were assigned to the control group only when they received standard therapy alone during the corresponding early-treatment window and had no record of probiotics or Tongli Gongxia Chinese medicine during that period. Patients exposed to probiotics alone or Tongli Gongxia Chinese medicine alone were not classified as controls for the primary matched comparison, thereby preserving a clear contrast between the combined exposure and standard therapy alone. Brief interruptions in therapy lasting ≤24 hours were treated as continuous treatment to account for temporary clinical circumstances such as intolerance or procedural interruptions that might not substantially alter the overall therapeutic exposure.

For patients who received probiotics or Tongli Gongxia Chinese medicine initiated after the 72-hour window, detailed timing information was recorded and incorporated into sensitivity analyses examining the impact of treatment timing on outcomes. The specific formulations, dosing regimens, routes of administration, and duration of therapy were systematically extracted from medication administration records and documented for each patient when available. In the matched control cohort, the early-treatment exposure distribution was probiotics alone 0/100, Tongli Gongxia Chinese medicine alone 0/100, and neither component 100/100.

### 2.3 Interventions and standard therapy

#### 2.3.1 Probiotic regimen

The probiotic intervention consisted of multi-strain formulations containing combinations of Lactobacillus acidophilus, Lactobacillus rhamnosus, Bifidobacterium animalis, and Bifidobacterium bifidum. These strains were used in routine clinical care because of documented colonization capabilities, safety profiles in critically ill patients, and potential relevance to SCFA-related metabolic pathways. The probiotics were administered either orally for patients capable of swallowing or via nasojejunal tube for those requiring enteral access, with each dose containing at least 1×10¹^0^ colony-forming units (CFU) of total viable organisms. The standard regimen consisted of 2 to 3 doses per day, with the typical total daily dose ranging from 2×10¹^0^ to 3×10¹^0^ CFU. Treatment was continued for a minimum of 3 days and typically extended throughout the period of active inflammatory response and barrier dysfunction, with median treatment duration of 7 to 10 days.

Detailed records were maintained for each patient regarding the specific probiotic product used, manufacturer, batch number when documented, route of administration, dose modifications, reasons for treatment discontinuation, and any adverse events temporally associated with probiotic use. Because these data reflected real-world clinical practice, variation in commercial product, viable count stability, excipients, and delivery route was considered part of the exposure heterogeneity and is acknowledged as a reproducibility limitation. A structured summary of probiotic strains, total daily dose, administration routes, and distribution of key exposure characteristics has been added as **Supplementary Table 1** to describe the major sources of exposure heterogeneity and support interpretation of reproducibility.

#### 2.3.2 Tongli Gongxia Chinese medicine

The Tongli Gongxia intervention was based on the classical Dachengqi Decoction formula, which comprises four principal herbs: Rheum palmatum (rhubarb), Magnolia officinalis (magnolia bark), Citrus aurantium (bitter orange), and Natrii Sulfas (mirabilite). The basic formula followed traditional proportions with rhubarb 9–15 g, magnolia bark 9–12 g, bitter orange 9–12 g, and mirabilite 6–12 g. In accordance with routine TCM practice, individual prescriptions could be modified within institutional protocols according to syndrome differentiation and gastrointestinal tolerance.

The herbal formulation was prepared according to standardized institutional protocols, either as traditional decoctions for oral administration or as granule concentrates dissolved in water for administration through nasojejunal tubes. For patients unable to take oral medications, the formula could be administered rectally as an enema. The initial dose was administered within 72 hours of admission, consistent with TCM principles emphasizing early intervention.

Comprehensive documentation was maintained for each patient regarding the specific herbal composition, dosage amounts for each ingredient, frequency of administration, route of delivery, start and stop dates, and any modifications made during the treatment course. Any adverse events or intolerance reactions such as excessive diarrhea, abdominal cramping, or electrolyte disturbances were systematically recorded. The real-world flexibility of syndrome-differentiated herbal prescription was not used to define separate causal contrasts and is discussed as a factor that may limit reproducibility and external validity. Because daily syndrome-differentiated modifications were recorded in legacy electronic medical records in an unstructured manner, complete patient-by-patient lists of all daily herbal modifications were not consistently retrievable. **Supplementary Table 1** therefore provides a structured summary of intervention characteristics and exposure heterogeneity, including core formula components, dose ranges, route distributions, and the most common syndrome-based additions.

#### 2.3.3 Standard therapy

Standard management included several key components implemented in a protocol-driven manner. Fluid resuscitation was initiated immediately upon diagnosis using goal-directed therapy with crystalloid solutions, typically lactated Ringer’s solution, targeting specific hemodynamic endpoints such as mean arterial pressure ≥65 mmHg, urine output ≥0.5 mL/kg/hour, and normalization of serum lactate. Pain management was achieved through multimodal analgesia including patient-controlled analgesia with opioids when appropriate.

Nutritional support represented a critical component of standard therapy, with institutional protocols strongly favoring early enteral nutrition (EN) initiated within 48 hours of admission when feasible. Enteral nutrition was delivered preferentially via nasojejunal tubes positioned under fluoroscopic or endoscopic guidance. The EN protocol followed a gradual escalation approach starting with trophic feeds at 10–20 mL/hour and advancing as tolerated. For patients in whom EN was contraindicated or not tolerated, parenteral nutrition was initiated.

Organ support measures were implemented according to standardized critical care protocols. Patients with respiratory failure received mechanical ventilation with lung-protective strategies. Those with acute kidney injury requiring renal replacement therapy received continuous renal replacement therapy (CRRT). Hemodynamic support with vasopressors and inotropes was provided according to established algorithms. Broad-spectrum antibiotics were administered according to institutional antimicrobial stewardship guidelines.

Additional interventions recorded as covariates included the use of proton pump inhibitors for stress ulcer prophylaxis, prokinetic agents to facilitate enteral feeding tolerance, and specific interventions for local complications such as interventional or surgical drainage procedures. The timing, duration, and specific characteristics of each of these interventions were systematically extracted from medical records to enable comprehensive adjustment in statistical models.

### 2.4 Outcome measures and assessment

#### 2.4.1 Primary outcomes

The primary outcomes of this study were the changes from baseline to day 7 in four serum biomarkers of intestinal barrier integrity: DAO, D-lactate, LPS, and I-FABP. These biomarkers were selected based on their established roles as indicators of enterocyte damage, bacterial translocation, and epithelial permeability. Baseline values were defined as measurements obtained within 24 hours of hospital admission. Day 7 values were obtained at 7 ± 1 days after admission.

The delta values (Δ) were calculated as day 7 value minus baseline value for each biomarker, with negative values indicating improvement (reduction) in barrier dysfunction markers. DAO was measured using enzyme-linked immunosorbent assay (ELISA) with results reported in units per liter (U/L). D-lactate was quantified using enzymatic spectrophotometric methods with results in millimoles per liter (mmol/L). LPS was measured using the Limulus amebocyte lysate (LAL) assay or assessed through LPS-binding protein (LBP) when direct measurement was not feasible, with results expressed in endotoxin units per milliliter (EU/mL). I-FABP was quantified using ELISA with results in picograms per milliliter (pg/mL).

#### 2.4.2 Secondary outcomes

Secondary outcomes encompassed multiple domains including metabolic, infectious, clinical, and safety endpoints. Fecal SCFA concentrations were measured at day 7 using gas chromatography-mass spectrometry (GC-MS) or gas chromatography with flame ionization detection (GC-FID). The SCFA panel included acetate (C2), propionate (C3), butyrate (C4), isobutyrate, valerate (C5), and isovalerate, with results reported as micromoles per gram of wet stool weight (μmol/g). Admission fecal SCFA samples were not available in the routine clinical archive; therefore, SCFA outcomes were prespecified as day 7 between-group comparisons rather than baseline-to-day 7 changes.

Infectious complications were assessed through systematic review of clinical, microbiological, and radiological data. Infected pancreatic necrosis (IPN) was defined according to consensus criteria as documented pancreatic necrosis with either positive cultures from fine-needle aspiration or surgical specimens, or strong clinical evidence of infection leading to antimicrobial therapy or invasive intervention. The diagnosis required multidisciplinary agreement.

Persistent organ failure was defined according to the Modified Marshall scoring system as failure of one or more organ systems persisting for more than 48 hours. ICU length of stay was calculated from ICU admission to discharge or death, expressed in days. Duration of mechanical ventilation was recorded in days for patients requiring invasive ventilatory support. The 28-day mortality was assessed as all-cause mortality occurring within 28 days of hospital admission.

Safety outcomes were comprehensively monitored and included diarrhea (defined as three or more loose stools per day persisting for at least 48 hours), abdominal distension, suspected probiotic-associated bloodstream infections, and electrolyte disturbances potentially related to Tongli Gongxia therapy. Any serious adverse events judged possibly related to the study interventions were systematically documented.

### 2.5 Sample collection and laboratory procedures

Blood samples for intestinal barrier biomarkers were collected at baseline, day 3, and day 7. Samples were processed using standard phlebotomy, centrifuged to obtain serum, and stored at -80 °C until batch analysis. DAO, D-lactate, LPS, LBP, and I-FABP were measured using commercially available ELISA kits or enzymatic assays following manufacturer protocols. All assays included quality control measures with calibration standards and quality control materials at multiple levels to ensure accuracy and reproducibility.

Fecal samples were collected at day 7 and immediately processed or stored at -80 °C. SCFAs were extracted using acidified water with internal standards, followed by centrifugation and acidification. Analysis was performed using GC-MS or GC-FID with appropriate capillary columns and temperature programs. Calibration curves were established using commercial standards, and quality control samples verified analytical performance. Results were expressed as micromoles per gram of wet fecal weight. Because retained admission fecal samples were unavailable, paired ΔSCFA values were not calculated.

### 2.6 Covariates and potential confounders

Demographic variables including age, sex, and BMI were collected. Acute pancreatitis etiology was classified as gallstone-induced, alcohol-induced, hypertriglyceridemia-induced, or other/idiopathic based on clinical history and laboratory findings. Disease severity was assessed using APACHE II and SOFA scores, along with admission lactate and CRP levels as markers of metabolic derangement and inflammation. Comorbidity burden was assessed through systematic review, documenting conditions including diabetes mellitus, chronic kidney disease, chronic liver disease, cardiovascular disease, and chronic obstructive pulmonary disease.

Temporal data regarding care delivery were extracted, including time from admission to treatment initiation, and timing and duration of key interventions such as enteral nutrition, antibiotics, vasopressor support, mechanical ventilation, and renal replacement therapy. Early enteral nutrition was defined as initiation within 48 hours. Antibiotic therapy details including specific agents and spectrum of coverage were documented. For patients undergoing invasive procedures such as ERCP, percutaneous drainage, or surgery, the timing and type were recorded. This granular temporal data facilitated adjustment for confounding and assessment of time-varying effects.

### 2.7 Propensity score matching and missing data handling

Propensity score matching was employed to reduce confounding and create balanced groups. The propensity score was estimated using multivariable logistic regression including baseline covariates such as demographics, etiology, severity scores, lactate, CRP, comorbidities, early enteral nutrition, antibiotic use, organ support, and calendar year. Patients were matched 1:1 using greedy nearest-neighbor algorithm with a 0.2 standard deviation caliper without replacement. Matching quality was assessed through standardized mean differences and propensity score distribution comparisons between groups.

Missing data extent and patterns were systematically evaluated. Little’s MCAR test was performed to assess missing patterns. For variables with 5-30% missing data, multiple imputation using chained equations (MICE) was employed to generate 10 imputed datasets. Continuous variables were imputed using predictive mean matching, while binary and categorical variables used logistic or multinomial regression. Imputation quality was assessed through distribution comparisons and convergence checks. Results were combined using Rubin’s rules.

### 2.8 Statistical analysis

Continuous variables were presented as mean ± SD or median with IQR based on normality assessment, while categorical variables were shown as frequencies and percentages. Baseline comparisons used t-tests, Mann-Whitney U tests, or chi-square tests as appropriate. Primary outcomes were analyzed using linear mixed-effects models with treatment group, time, and group-by-time interaction as fixed effects, adjusted for confounders. Secondary outcomes were analyzed using appropriate methods based on outcome type: t-tests or Mann-Whitney U tests for continuous outcomes, and chi-square or Fisher’s exact tests for binary outcomes.

Multivariable linear regression models assessed the independent association between combination therapy and barrier biomarker changes, adjusting for confounders selected *a priori* including APACHE II score, early enteral nutrition, antibiotic use, admission lactate, treatment timing, and laboratory batch effects. Multiple imputation was performed and results were pooled according to Rubin’s rules. Multiple comparison adjustment used the Benjamini-Hochberg procedure to control false discovery rate at 5%.

Sensitivity analyses included subgroup analyses stratified by etiology, early enteral nutrition status, and antibiotic use to explore potential effect modification. Analyses were also repeated after excluding patients who underwent surgical or interventional procedures within 48 hours of admission. Inverse probability of treatment weighting (IPTW) using the propensity score was employed as an alternative to matching. E-value calculations were performed to assess the potential impact of unmeasured confounding.

The retrospective study identified 240 eligible patients from 2019-2024, with propensity score matching yielding 200 patients (100 per group). The power assessment indicated approximately 90% power for primary outcomes (detecting a difference of 2.0 U/L for DAO with common SD of 4.5 U/L, corresponding to Cohen’s d ≈ 0.44). For ICU length of stay, the study had approximately 75-80% power to detect a median difference of 2 days. Binary outcomes such as infected pancreatic necrosis with expected incidences of approximately 25% in controls and 15% in intervention (10% absolute risk reduction) had approximately 50-60% power and were therefore interpreted cautiously.

All analyses were performed using R statistical software version 4.2 or higher with relevant packages including MatchIt for propensity score matching, lme4 for mixed-effects models, mice for multiple imputation, and ggplot2 for graphics. Statistical significance was assessed using two-sided tests with α=0.05. A detailed statistical analysis plan was documented prior to data analysis.

## 3 Results

### 3.1 Patient flow and baseline characteristics

**Figure 1:**
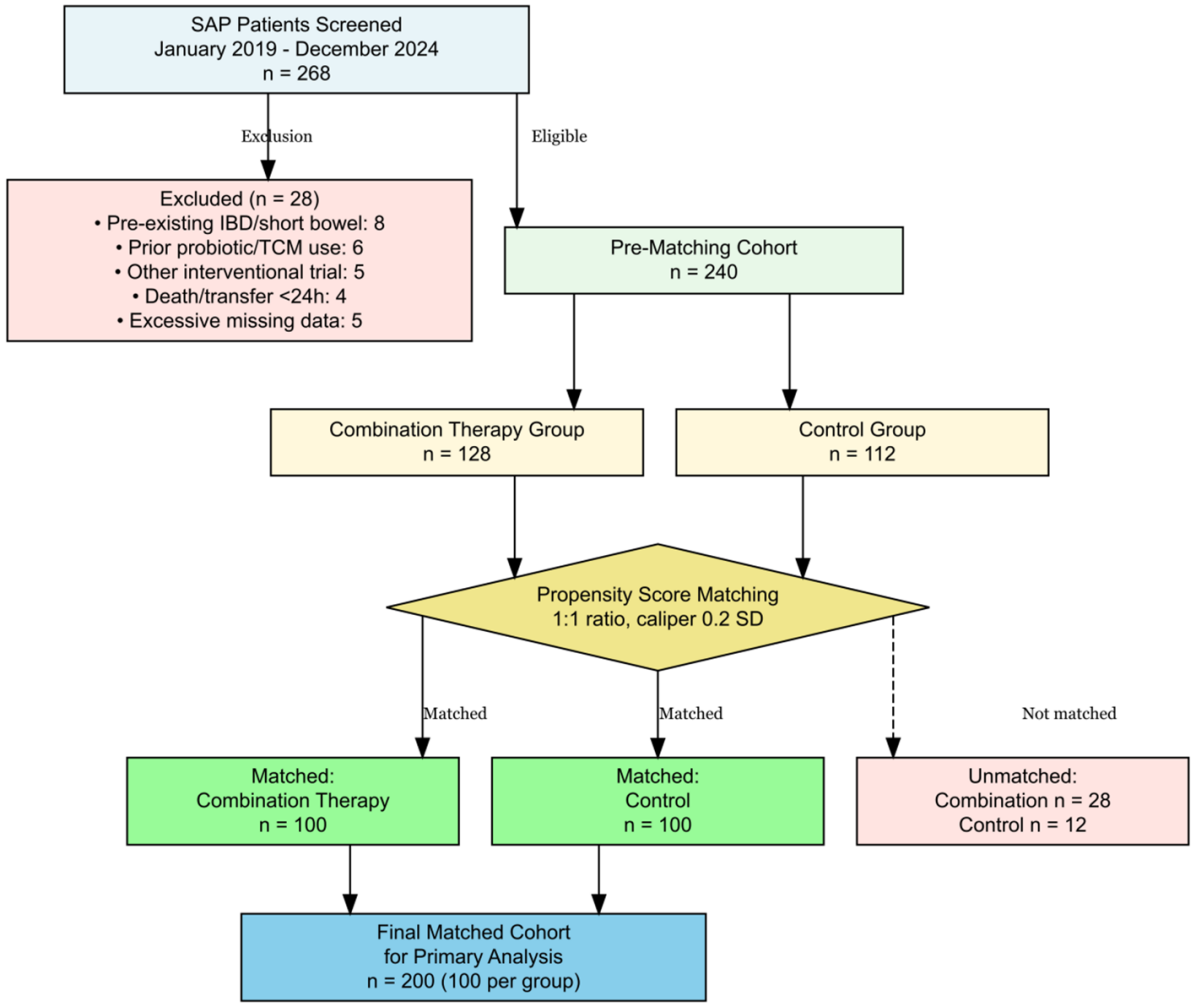
Patient flow diagram.

Between January 1, 2019, and December 31, 2024, a total of 268 patients with SAP were screened for eligibility. Of these, 28 patients were excluded based on the predefined criteria: 8 had pre-existing inflammatory bowel disease or short bowel syndrome, 6 had received probiotics or Tongli Gongxia formulations within one month prior to admission, 5 were participating in other interventional trials, 4 died or were transferred within 24 hours of admission, and 5 had excessive missing data that precluded reliable analysis (**Figure 1**). The remaining 240 patients constituted the pre-matching cohort, comprising 128 patients in the combination therapy group and 112 patients in the control group.

After propensity score matching using a 1:1 ratio with a caliper of 0.2 standard deviations of the logit of the propensity score, 200 patients (100 in each group) were successfully matched and included in the primary analysis. The matched control group was composed entirely of patients receiving standard therapy alone during the early-treatment window: 0 received probiotics alone, 0 received Tongli Gongxia Chinese medicine alone, and 100 received neither component. The matching process achieved excellent balance across baseline covariates, with all standardized mean differences less than 0.1 (**Table 1**). The matched groups showed similar distributions of age (mean 52.3 ± 13.2 years in combination group vs. 51.8 ± 12.9 years in control group, SMD = 0.04), sex (63% male in both groups, SMD = 0.00), BMI (25.7 ± 3.8 vs. 25.4 ± 3.6 kg/m², SMD = 0.08), and disease severity scores (APACHE II 12.4 ± 4.2 vs. 12.1 ± 4.0, SMD = 0.07; SOFA 5.8 ± 2.6 vs. 5.6 ± 2.5, SMD = 0.08). The distribution of pancreatitis etiology was also balanced, with gallstone-induced cases representing 42% and 44%, alcohol-induced 31% and 29%, hypertriglyceridemia-induced 18% and 19%, and other causes 9% and 8% in the combination and control groups, respectively (p=0.93). Baseline values of the four barrier biomarkers showed no significant differences: DAO (14.8 ± 5.2 vs. 14.5 ± 5.0 U/L, p=0.69), D-lactate (6.8 ± 2.4 vs. 6.7 ± 2.3 mmol/L, p=0.76), LPS (0.42 ± 0.18 vs. 0.41 ± 0.17 EU/mL, p=0.70), and I-FABP (1245 ± 428 vs. 1218 ± 411 pg/mL, p=0.64).

**Table 1:** Baseline characteristics of matched cohort.

Characteristic	Combination therapy (n=100)	Control (n=100)	SMD	P-value
Demographics				
Age, years (mean ± SD)	52.3 ± 13.2	51.8 ± 12.9	0.04	0.78
Male sex, n (%)	63 (63.0)	63 (63.0)	0.00	1.00
Body mass index, kg/m² (mean ± SD)	25.7 ± 3.8	25.4 ± 3.6	0.08	0.54
Etiology, n (%)				0.93
Gallstone	42 (42.0)	44 (44.0)	0.04	
Alcohol	31 (31.0)	29 (29.0)	0.04	
Hypertriglyceridemia	18 (18.0)	19 (19.0)	0.03	
Other	9 (9.0)	8 (8.0)	0.04	
Severity Scores				
APACHE II (mean ± SD)	12.4 ± 4.2	12.1 ± 4.0	0.07	0.60
SOFA (mean ± SD)	5.8 ± 2.6	5.6 ± 2.5	0.08	0.57
Laboratory Values				
Admission lactate, mmol/L (mean ± SD)	3.2 ± 1.6	3.1 ± 1.5	0.06	0.63
Admission CRP, mg/L (median [IQR])	142 [98-198]	138 [95-192]	0.07	0.68
Baseline Barrier Biomarkers				
DAO, U/L (mean ± SD)	14.8 ± 5.2	14.5 ± 5.0	0.06	0.69
D-lactate, mmol/L (mean ± SD)	6.8 ± 2.4	6.7 ± 2.3	0.04	0.76
LPS, EU/mL (mean ± SD)	0.42 ± 0.18	0.41 ± 0.17	0.06	0.70
I-FABP, pg/mL (mean ± SD)	1245 ± 428	1218 ± 411	0.07	0.64
Early Management				
Early EN (≤48h), n (%)	78 (78.0)	76 (76.0)	0.05	0.73
Broad-spectrum antibiotics (≤72h), n (%)	64 (64.0)	62 (62.0)	0.04	0.77
Mechanical ventilation (≤72h), n (%)	32 (32.0)	30 (30.0)	0.04	0.76
Vasopressor support (≤72h), n (%)	28 (28.0)	26 (26.0)	0.05	0.75
Comorbidities, n (%)				
Diabetes mellitus	24 (24.0)	22 (22.0)	0.05	0.74
Chronic kidney disease	12 (12.0)	11 (11.0)	0.03	0.82
Chronic liver disease	8 (8.0)	9 (9.0)	0.04	0.80
Cardiovascular disease	18 (18.0)	17 (17.0)	0.03	0.85

Matched control exposure during the early-treatment window: probiotics alone 0/100, Tongli Gongxia alone 0/100, and neither component 100/100. Abbreviations: SMD, standardized mean difference; APACHE, Acute Physiology and Chronic Health Evaluation; SOFA, Sequential Organ Failure Assessment; CRP, C-reactive protein; DAO, diamine oxidase; LPS, lipopolysaccharide; I-FABP, intestinal fatty acid-binding protein; EN, enteral nutrition; IQR, interquartile range.

### 3.2 Primary outcomes: intestinal barrier biomarker changes

**Figure 2:**
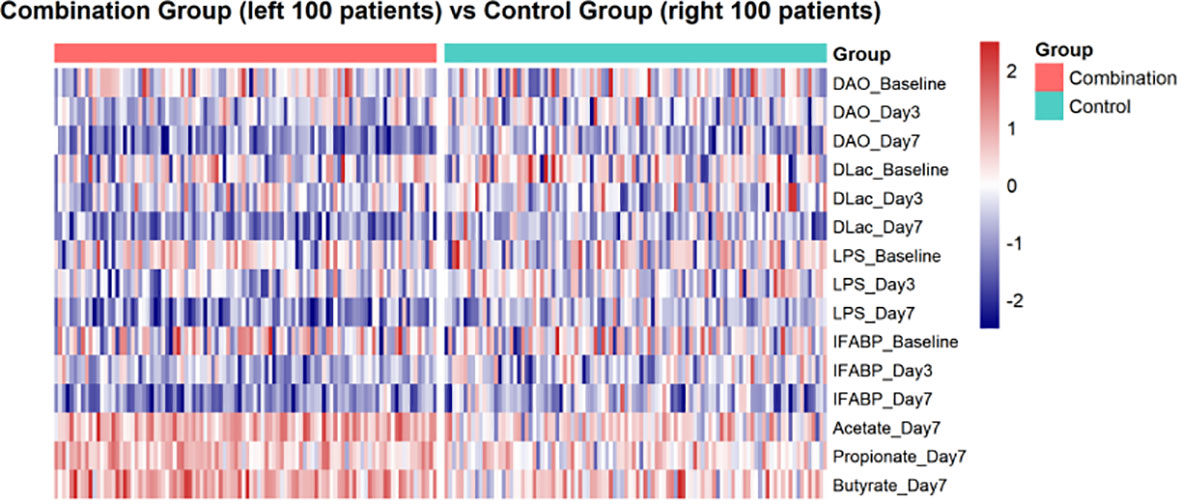
Heatmap of standardized barrier biomarkers and day 7 SCFA concentrations.

Linear mixed-effects models demonstrated significant group-by-time interactions for all four barrier biomarkers (p<0.001 for DAO, D-lactate, and LPS; p=0.002 for I-FABP), indicating that the temporal patterns of biomarker change differed between the two groups (**Table 2**, **Figure 2**). At day 3, modest differences were beginning to emerge, with the combination group showing slightly greater reductions compared to control, although not all differences reached statistical significance at this early timepoint. By day 7, the between-group differences had become more pronounced.

**Table 2:** Changes in intestinal barrier biomarkers over time.

Biomarker	Time point	Combination therapy (n=100)	Control (n=100)	Between-group difference (95% CI)	P-value
DAO (U/L)					
	Baseline	14.8 ± 5.2	14.5 ± 5.0	–	0.69
	Day 3	11.6 ± 4.8	12.4 ± 5.1	-0.8 (-2.1 to 0.5)	0.22
	Day 7	9.0 ± 4.2	11.3 ± 4.9	-2.3 (-3.6 to -1.0)	<0.001
	Δ Baseline to Day 7	-5.8 ± 3.9	-3.2 ± 4.2	-2.6 (-3.8 to -1.4)	<0.001*
D-lactate (mmol/L)					
	Baseline	6.8 ± 2.4	6.7 ± 2.3	–	0.76
	Day 3	5.2 ± 2.1	5.6 ± 2.2	-0.4 (-1.0 to 0.2)	0.18
	Day 7	3.4 ± 1.8	4.6 ± 2.0	-1.2 (-1.7 to -0.7)	<0.001
	Δ Baseline to Day 7	-3.4 ± 1.8	-2.1 ± 2.0	-1.3 (-1.9 to -0.7)	<0.001*
LPS (EU/mL)					
	Baseline	0.42 ± 0.18	0.41 ± 0.17	–	0.70
	Day 3	0.32 ± 0.15	0.35 ± 0.16	-0.03 (-0.07 to 0.01)	0.13
	Day 7	0.21 ± 0.12	0.29 ± 0.14	-0.08 (-0.12 to -0.04)	<0.001
	Δ Baseline to Day 7	-0.21 ± 0.13	-0.12 ± 0.14	-0.09 (-0.13 to -0.05)	<0.001*
I-FABP (pg/mL)					
	Baseline	1245 ± 428	1218 ± 411	–	0.64
	Day 3	892 ± 376	986 ± 398	-94 (-198 to 10)	0.08
	Day 7	617 ± 324	806 ± 368	-189 (-289 to -89)	<0.001
	Δ Baseline to Day 7	-628 ± 345	-412 ± 362	-216 (-328 to -104)	<0.001*

Values are presented as mean ± SD. Δ represents change from baseline (Day 7 value minus baseline value).

Remains statistically significant after Benjamini-Hochberg FDR correction (adjusted p<0.002 for all four primary outcomes). DAO, diamine oxidase; LPS, lipopolysaccharide; I-FABP, intestinal fatty acid-binding protein; CI, confidence interval; FDR, false discovery rate.

For DAO, the combination group demonstrated a mean decrease of 5.8 ± 3.9 U/L from baseline to day 7, compared with 3.2 ± 4.2 U/L in the control group, representing an additional mean reduction of 2.6 U/L (95% CI 1.4 to 3.8 U/L, p<0.001). D-lactate showed a similar pattern, with the combination group reducing by 3.4 ± 1.8 mmol/L versus 2.1 ± 2.0 mmol/L in controls, for a between-group difference of 1.3 mmol/L (95% CI 0.7 to 1.9 mmol/L, p<0.001). LPS decreased by 0.21 ± 0.13 EU/mL in the combination group compared with 0.12 ± 0.14 EU/mL in controls, representing an additional reduction of 0.09 EU/mL (95% CI 0.05 to 0.13 EU/mL, p<0.001). I-FABP demonstrated a reduction of 628 ± 345 pg/mL in the combination group versus 412 ± 362 pg/mL in controls, with a between-group difference of 216 pg/mL (95% CI 104 to 328 pg/mL, p<0.001). These findings remained statistically significant after adjustment for multiple comparisons using the Benjamini-Hochberg procedure (all FDR-adjusted p<0.002) and should be interpreted as associations within a retrospective matched cohort.

### 3.3 Secondary outcomes: short-chain fatty acid metabolic profiles

**Figure 3:**
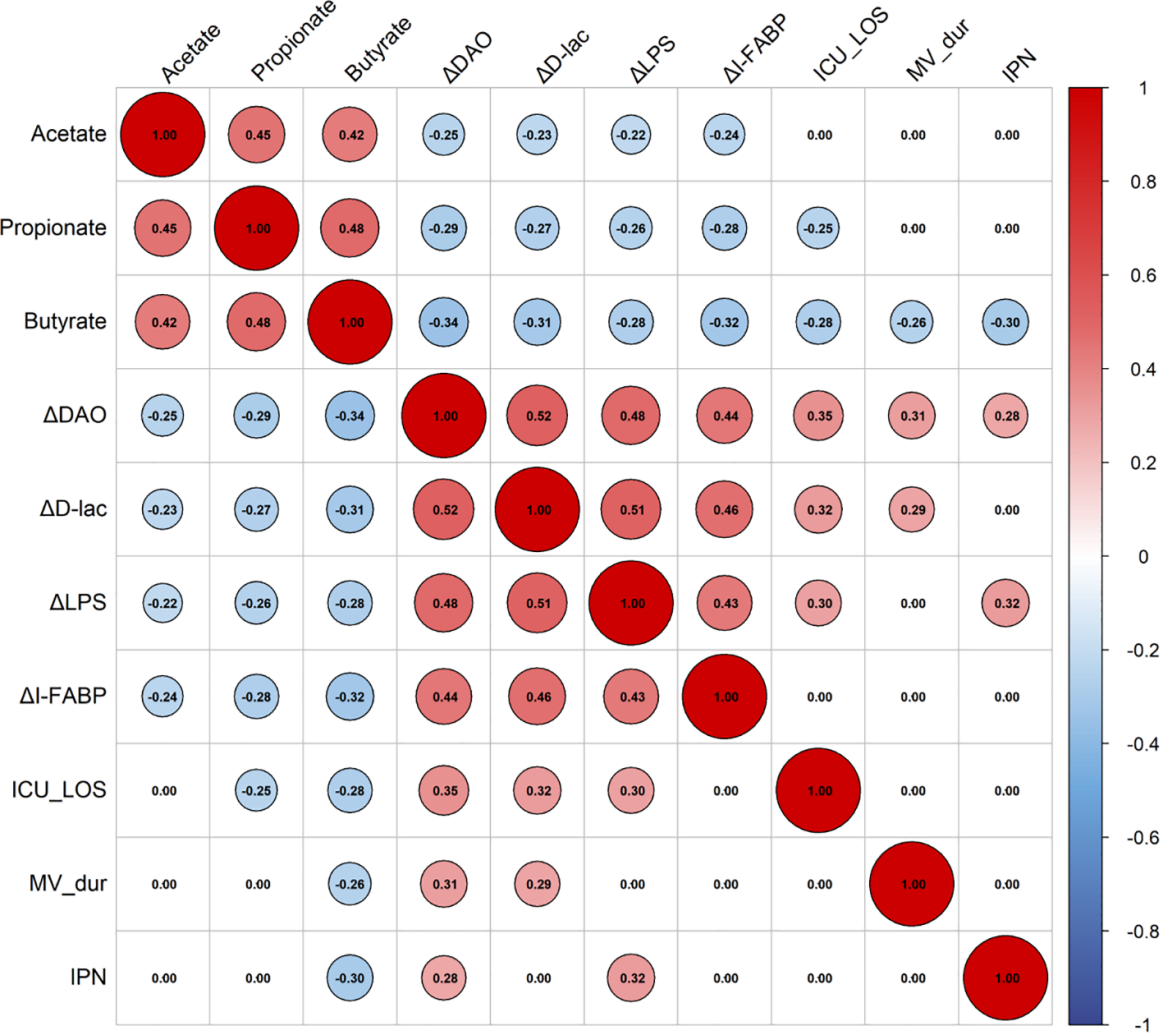
Correlation matrix of day 7 SCFA concentrations and barrier biomarker changes.

Fecal SCFA analysis at day 7 revealed higher concentrations of major SCFAs in the combination therapy group compared to controls (**Table 3**, **Figures 2**, **3**). Acetate, the most abundant SCFA, showed a median concentration of 58.3 (IQR 45.2-72.8) μmol/g in the combination group versus 47.6 (IQR 36.4-61.2) μmol/g in controls (p=0.001). Propionate concentrations were 21.4 (IQR 16.8-28.3) μmol/g versus 17.2 (IQR 12.9-23.6) μmol/g (p=0.002). Butyrate, which has been implicated in maintaining barrier integrity and modulating inflammation, demonstrated median levels of 14.6 (IQR 10.2-20.1) μmol/g in the combination group compared to 10.8 (IQR 7.4-15.3) μmol/g in controls (p=0.003). Because baseline fecal SCFA samples were unavailable, these results indicate higher day 7 concentrations and do not establish treatment-induced SCFA increases.

**Table 3:** Fecal short-chain fatty acid concentrations at day 7.

SCFA (μmol/g wet weight)	Combination therapy (n=100) median (IQR)	Control (n=100) median (IQR)	Difference	P-value
Major SCFAs				
Acetate (C2)	58.3 (45.2-72.8)	47.6 (36.4-61.2)	+10.7	0.001
Propionate (C3)	21.4 (16.8-28.3)	17.2 (12.9-23.6)	+4.2	0.002
Butyrate (C4)	14.6 (10.2-20.1)	10.8 (7.4-15.3)	+3.8	0.003
Branched-Chain SCFAs				
Isobutyrate	2.8 (2.1-3.9)	2.3 (1.6-3.2)	+0.5	0.02
Valerate (C5)	5.4 (3.8-7.6)	4.6 (3.1-6.8)	+0.8	0.08
Isovalerate	3.1 (2.3-4.2)	2.5 (1.8-3.6)	+0.6	0.03
Total SCFAs	105.6 (84.3-132.9)	85.0 (65.7-108.4)	+20.6	0.001

Values are median (interquartile range). P-values are from Mann-Whitney U tests. Because baseline fecal SCFA samples were unavailable, these values represent day 7 between-group comparisons and should not be interpreted as baseline-to-day 7 SCFA changes.

SCFA, short-chain fatty acid; IQR, interquartile range.

The branched-chain SCFAs, isobutyrate and isovalerate, which are products of protein fermentation, also showed modest but statistically significant elevations in the combination group: isobutyrate 2.8 (2.1-3.9) versus 2.3 (1.6-3.2) μmol/g (p=0.02) and isovalerate 3.1 (2.3-4.2) versus 2.5 (1.8-3.6) μmol/g (p=0.03). Valerate levels showed a similar trend (5.4 [3.8-7.6] vs. 4.6 [3.1-6.8] μmol/g, p=0.08), though this did not reach statistical significance. The total SCFA concentration, calculated as the sum of all measured SCFAs, was significantly higher in the combination group: 105.6 (IQR 84.3-132.9) versus 85.0 (IQR 65.7-108.4) μmol/g (p=0.001).

Correlation analysis revealed inverse relationships between day 7 SCFA concentrations and the magnitude of barrier biomarker reduction. Specifically, butyrate concentration showed a moderate negative correlation with ΔDAO (r=-0.34, p<0.001), ΔD-lactate (r=-0.31, p<0.001), and ΔLPS (r=-0.28, p=0.002) after FDR correction for multiple comparisons. Propionate demonstrated similar but slightly weaker correlations (r=-0.26 to -0.29, all p<0.01). These relationships were visualized using a correlation matrix diagram that highlighted associations between day 7 metabolic profiles and barrier biomarker changes (**Figure 3**), but they should not be interpreted as proof of a causal metabolic restoration pathway.

### 3.4 Multivariable models and robustness analyses

**Figure 4:**
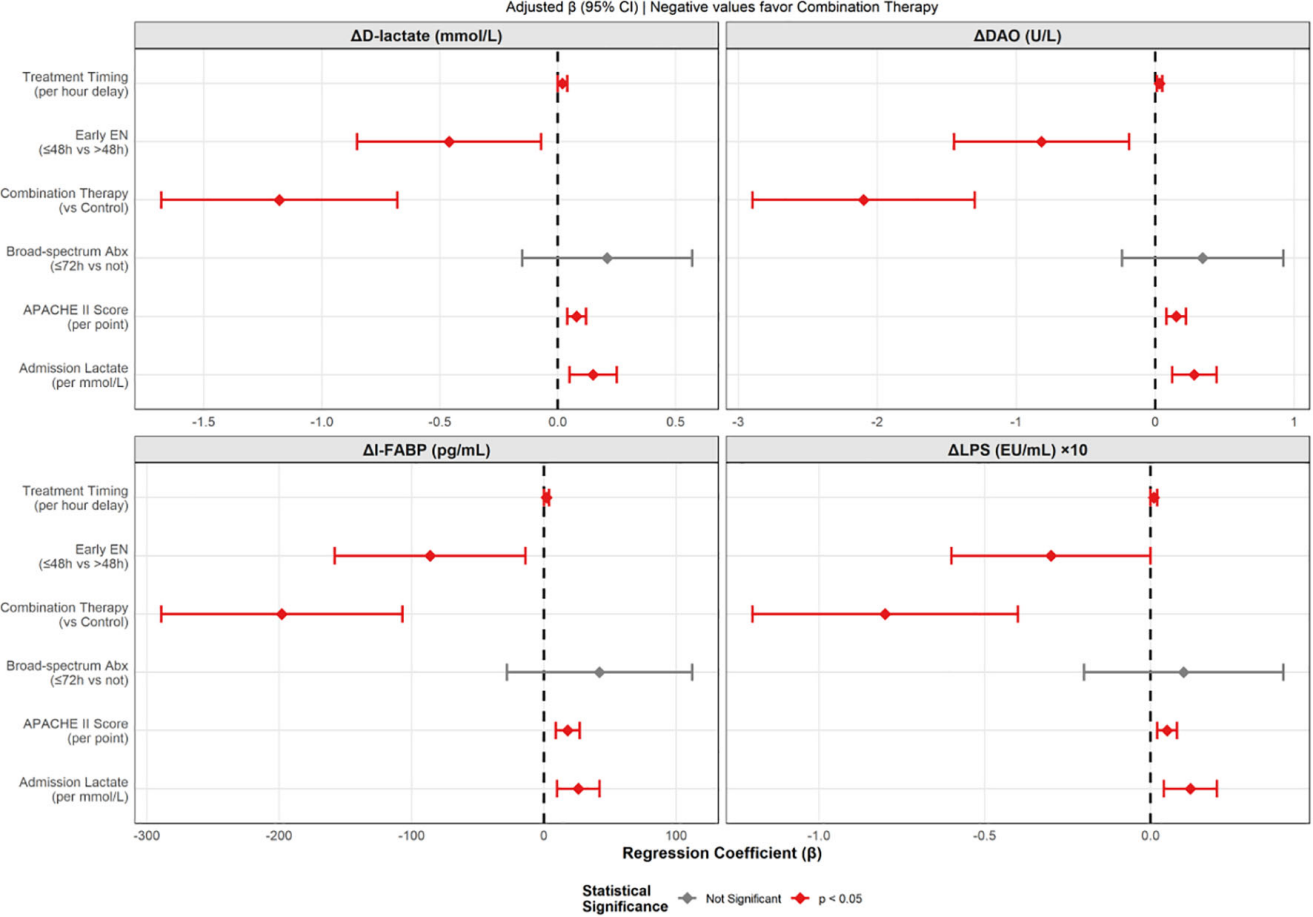
Forest plot of multivariable regression coefficients.

Multivariable linear regression models adjusting for disease severity, early enteral nutrition, antibiotic use, admission lactate, treatment timing, and laboratory batch effects confirmed the independent association between combination therapy and greater reductions in barrier biomarkers (**Table 4**, **Figure 4**). For ΔDAO, the adjusted regression coefficient for combination therapy was -2.10 U/L (95% CI -2.90 to -1.30, p<0.001), indicating that after accounting for measured confounders, the combination group was associated with a 2.10 U/L greater reduction in DAO compared to controls. Similar patterns were observed for ΔD-lactate (β=-1.18 mmol/L, 95% CI -1.68 to -0.68, p<0.001), ΔLPS (β=-0.08 EU/mL, 95% CI -0.12 to -0.04, p<0.001), and ΔI-FABP (β=-198 pg/mL, 95% CI -289 to -107, p<0.001).

**Table 4:** Multivariable regression models for changes in barrier biomarkers.

Variable	ΔDAO (U/L) β (95% CI)	ΔD-lactate (mmol/L) β (95% CI)	ΔLPS (EU/mL) β (95% CI)	ΔI-FABP (pg/mL) β (95% CI)
Combination therapy (vs. Control)	-2.10 (-2.90 to -1.30)***	-1.18 (-1.68 to -0.68)***	-0.08 (-0.12 to -0.04)***	-198 (-289 to -107)***
APACHE II score (per point)	+0.15 (+0.08 to +0.22)***	+0.08 (+0.04 to +0.12)**	+0.005 (+0.002 to +0.008)**	+18 (+9 to +27)***
Early EN (≤48h)	-0.82 (-1.45 to -0.19)*	-0.46 (-0.85 to -0.07)*	-0.03 (-0.06 to -0.00)*	-86 (-158 to -14)*
Broad-spectrum antibiotics (≤72h)	+0.34 (-0.24 to +0.92)	+0.21 (-0.15 to +0.57)	+0.01 (-0.02 to +0.04)	+42 (-28 to +112)
Admission lactate (per mmol/L)	+0.28 (+0.12 to +0.44)**	+0.15 (+0.05 to +0.25)**	+0.012 (+0.004 to +0.020)**	+26 (+10 to +42)**
Treatment initiation timing (per hour)	+0.03 (+0.01 to +0.05)*	+0.02 (+0.00 to +0.04)*	+0.001 (+0.000 to +0.002)*	+2 (+0 to +4)*
Model R²	0.42	0.39	0.35	0.38

β represents the regression coefficient (effect size) from multivariable linear regression models.

***P<0.001; **P<0.01; *P<0.05.

All models also adjusted for laboratory batch/platform (not shown). Negative β values for combination therapy indicate greater improvement (reduction) in biomarkers compared to control. DAO, diamine oxidase; LPS, lipopolysaccharide; I-FABP, intestinal fatty acid-binding protein; CI, confidence interval; APACHE, Acute Physiology and Chronic Health Evaluation; EN, enteral nutrition.

Among the covariates, APACHE II score showed a consistent association with attenuated biomarker improvement (higher scores associated with smaller reductions), suggesting that greater disease severity limited the potential for barrier recovery regardless of treatment. Early enteral nutrition was associated with enhanced biomarker improvement across all models (all p<0.05), supporting the importance of early nutritional support in SAP management. Interestingly, the use of broad-spectrum antibiotics within 72 hours showed variable associations across biomarkers, with some suggestion of attenuation of treatment effect for DAO and D-lactate, potentially reflecting antibiotic-associated dysbiosis effects.

Sensitivity analyses demonstrated consistency of findings across multiple analytical approaches. Subgroup analyses stratified by etiology revealed relatively consistent treatment effects across gallstone-induced, alcohol-induced, and hypertriglyceridemia-induced SAP, with no significant treatment-by-etiology interaction (p-values for interaction terms ranged from 0.24 to 0.68). Stratification by early enteral nutrition status suggested a potentially greater treatment benefit among patients who received early EN (p-interaction=0.08 for DAO), though this interaction did not reach statistical significance and should be interpreted cautiously. Exclusion of patients who underwent surgical or interventional procedures within 48 hours of admission (n=23) did not substantially alter the results, with point estimates changing by less than 10% and all conclusions remaining unchanged.

Analysis using IPTW as an alternative to matching produced similar results with slightly wider confidence intervals but consistent directionality and statistical significance. The E-value for the association between combination therapy and ΔDAO was 2.48, indicating that an unmeasured confounder would need to have a risk ratio of at least 2.48 with both treatment assignment and the outcome to fully explain away the observed association, suggesting robustness to unmeasured confounding of moderate strength.

### 3.5 Clinical outcomes and safety assessment

**Figure 5:**
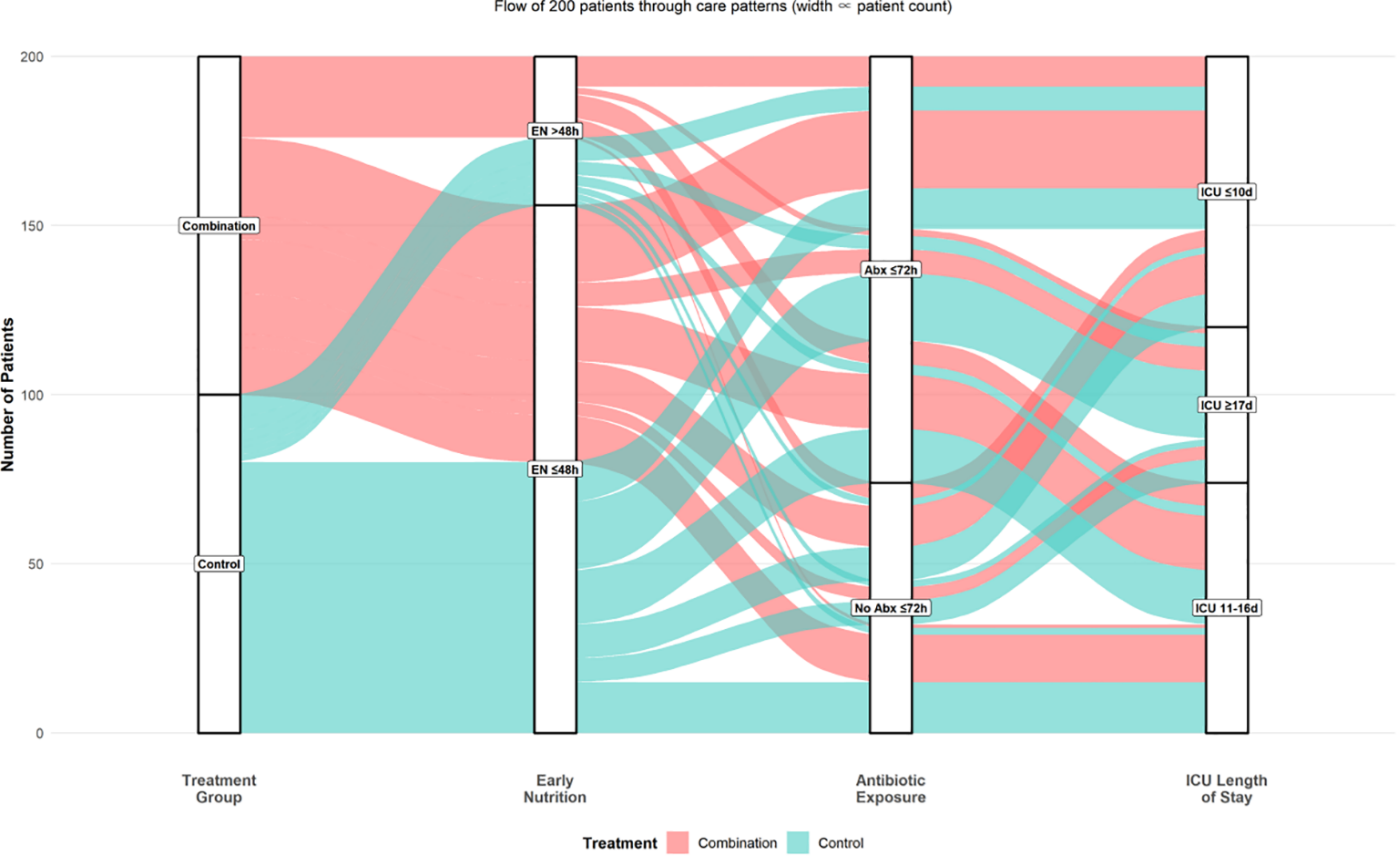
Sankey diagram of treatment pathways and ICU length-of-stay categories.

The combination therapy group demonstrated a shorter ICU length of stay compared to controls, with a median of 11 days (IQR 8-16) versus 13 days (IQR 9-19), representing a 2-day difference (p=0.03 by Mann-Whitney U test) (**Table 5**, **Figure 5**). Duration of mechanical ventilation showed a similar trend favoring the combination group, with median 6 days (IQR 3-10) versus 7 days (IQR 4-12), though this difference did not reach statistical significance (p=0.12). Hospital length of stay was also numerically shorter in the combination group (24 [IQR 18-32] vs. 27 [IQR 20-36] days, p=0.08). Thus, clinical outcome advantages were modest, with ICU length of stay being the only statistically significant clinical endpoint.

**Table 5:** Clinical outcomes and safety events.

Outcome	Combination therapy (n=100)	Control (n=100)	Effect estimate (95% CI)	P-value
Clinical Outcomes				
ICU length of stay, days (median [IQR])	11 (8-16)	13 (9-19)	Difference: -2 days	0.03
Mechanical ventilation duration, days (median [IQR])†	6 (3-10)	7 (4-12)	Difference: -1 day	0.12
Hospital length of stay, days (median [IQR])	24 (18-32)	27 (20-36)	Difference: -3 days	0.08
Infected pancreatic necrosis, n (%)	14 (14.0)	21 (21.0)	RD: -7% (-17% to 3%)	0.15
New persistent organ failure ≤28 days, n (%)	22 (22.0)	28 (28.0)	RD: -6% (-17% to 5%)	0.32
28-day mortality, n (%)	8 (8.0)	12 (12.0)	RD: -4% (-12% to 4%)	0.38
Safety Events				
Diarrhea (≥3 loose stools/day ≥48h), n (%)	28 (28.0)	18 (18.0)	RD: +10% (-1% to 21%)	0.09
Severe diarrhea requiring cessation, n (%)	3 (3.0)	1 (1.0)	RD: +2% (-2% to 6%)	0.62‡
Probiotic-associated bloodstream infection, n (%)	0 (0.0)	0 (0.0)	–	–
Clinically significant electrolyte disturbance, n (%)	5 (5.0)	2 (2.0)	RD: +3% (-2% to 8%)	0.45‡
Abdominal distension requiring intervention, n (%)	12 (12.0)	14 (14.0)	RD: -2% (-11% to 7%)	0.68

†Among patients requiring mechanical ventilation (n=32 in combination group, n=30 in control group).

‡Fisher’s exact test used due to expected cell counts <5. IQR, interquartile range; ICU, intensive care unit; RD, risk difference; CI, confidence interval.

P-values from Mann-Whitney U tests (continuous outcomes) or chi-square/Fisher’s exact tests (categorical outcomes).

Several adverse prognostic outcomes relevant to SAP progression were compared between groups (Huang et al., 2022). The incidence of infected pancreatic necrosis showed a trend toward reduction in the combination group (14% vs. 21%, risk difference -7%, 95% CI -17% to 3%, p=0.15), though this did not reach statistical significance. Similarly, new persistent organ failure within 28 days occurred in 22% of combination therapy patients versus 28% of controls (risk difference -6%, 95% CI -17% to 5%, p=0.32). The 28-day mortality rate was 8% in the combination group compared to 12% in the control group (risk difference -4%, 95% CI -12% to 4%, p=0.38). These findings were not statistically significant, the study was not powered to detect differences in these relatively infrequent outcomes with adequate precision, and the observed trends should be considered exploratory.

Safety analysis revealed no episodes of culture-confirmed probiotic-associated bloodstream infections in either group. Diarrhea meeting the predefined criteria occurred in 28% of combination therapy patients versus 18% of controls (p=0.09), representing a modest increase that did not reach statistical significance. Most diarrhea cases in the combination group were mild to moderate in severity and self-limited or responsive to dose adjustment of the Tongli Gongxia formula. Severe diarrhea requiring cessation of therapy occurred in only 3 patients (3%) in the combination group. Clinically significant electrolyte disturbances potentially attributable to the laxative effects of the Chinese medicine occurred in 5 patients (5%) in the combination group versus 2 patients (2%) in the control group (p=0.45), with all cases successfully managed through electrolyte monitoring and supplementation. Because diarrhea and fluid shifts may be clinically important in vulnerable SAP patients, this safety signal requires careful monitoring in future prospective studies. There were no episodes of intestinal perforation, severe hemorrhage, or other serious adverse events judged to be related to the combination therapy.

## 4 Discussion

This single-center retrospective cohort study suggests that the addition of probiotics combined with Tongli Gongxia Chinese medicine to standard therapy is associated with greater improvements in intestinal barrier function biomarkers and higher day 7 fecal SCFA concentrations in patients with SAP. After propensity score matching and multivariable adjustment, the combination therapy group demonstrated consistently greater reductions in four validated markers of enterocyte damage and barrier dysfunction (DAO, D-lactate, LPS, and I-FABP) at day 7 compared to controls receiving standard therapy alone. Concurrently, patients receiving combination therapy exhibited higher day 7 concentrations of beneficial SCFAs, particularly acetate, propionate, and butyrate. These metabolic and barrier findings were accompanied by a shorter ICU length of stay, whereas differences in mechanical ventilation duration, hospital length of stay, infected pancreatic necrosis, persistent organ failure, and 28-day mortality were not statistically significant. The findings should therefore be interpreted as hypothesis-generating associations rather than definitive evidence of efficacy or causality.

The mechanistic rationale for combining probiotics with Tongli Gongxia herbs rests on potentially complementary actions on intestinal homeostasis. SAP induces disturbances in intestinal motility, microbial ecology, and barrier integrity through multiple pathways including splanchnic hypoperfusion, inflammatory cytokine release, and stress-induced alterations in the intestinal microenvironment (Mederos et al., 2021; Iannuzzi et al., 2022). The resulting stasis and dysbiosis may create conditions favoring pathogenic bacterial overgrowth, particularly in the small intestine, while diminishing populations of beneficial commensal organisms that produce SCFAs and maintain colonization resistance (Gerritsen et al., 2011; Tan et al., 2015). Direct supplementation with probiotics aims to support beneficial microbial populations and their metabolic activities, but the efficacy of this approach may be limited if the underlying intestinal environment remains inhospitable to colonization (Gou et al., 2014; Kothari et al., 2019).

The Tongli Gongxia principle, operationalized through formulations such as Dachengqi Decoction, may address several of these environmental factors. The constituent herbs exert prokinetic effects through mechanisms including stimulation of intestinal smooth muscle contractility, modulation of neurotransmitter release, and alteration of water and electrolyte flux into the lumen (Zhang et al., 2015; Hu et al., 2018). By enhancing intestinal transit and reducing stasis, these herbs may facilitate the clearance of pathogenic organisms and toxic metabolites while limiting proximal bacterial overgrowth that can occur in SAP (Yang et al., 2021). Additionally, compounds in rhubarb and other herbs demonstrate antimicrobial or microbiota-modulating activities in experimental contexts, although the present study did not directly measure gut microbial composition (Gao et al., 2021; Dai et al., 2023). Accordingly, statements regarding ecological rebalancing or enrichment of SCFA-producing bacteria remain mechanistic hypotheses rather than directly demonstrated findings.

The higher day 7 butyrate concentrations observed in the combination therapy group are noteworthy given butyrate’s established roles in intestinal homeostasis. Butyrate serves as an energy source for colonocytes and can enhance barrier function by influencing tight junction proteins including occludin, claudin-1, and zonula occludens-1 (Liu et al., 2018; Parada Venegas et al., 2019). It also exerts anti-inflammatory effects through mechanisms including inhibition of NF-κB signaling, promotion of regulatory T cell differentiation, and modulation of inflammasome activity (Correa-Oliveira et al., 2016). The moderate inverse correlations between butyrate concentrations and barrier biomarker changes (r=-0.34 for DAO) are compatible with a metabolite-barrier relationship described in experimental models (Fukuda et al., 2011). However, because fecal SCFAs were measured only at day 7 and baseline SCFA levels were unavailable, these correlations cannot establish paired metabolic recovery or prove that the intervention increased SCFA production from baseline.

The association between combination therapy and shorter ICU length of stay, while secondary to our mechanistic outcomes, has potential clinical relevance. ICU length of stay is a clinically meaningful endpoint that integrates multiple aspects of recovery including resolution of organ dysfunction, tolerance of enteral nutrition, and control of complications (Petrov et al., 2010). The 2-day median difference observed in this cohort is clinically interesting given the high costs and risks associated with prolonged ICU care. However, other clinical endpoints were not statistically significant, and the study had limited power for relatively infrequent outcomes. Previous randomized trials of probiotics alone in SAP have shown mixed results on clinical outcomes, with some suggesting harm (Besselink et al., 2008; Barraud et al., 2013). Our findings therefore support further prospective evaluation of this combined real-world strategy, but they do not establish superiority over either component alone.

The safety profile of the combination therapy in our cohort is reassuring but requires cautious interpretation. We observed no cases of probiotic-associated bloodstream infections, a concern that has limited enthusiasm for probiotic use in critically ill patients following reports of probiotic-associated complications in earlier studies (Barraud et al., 2013; Doron and Snydman, 2015). Several factors may have contributed to the favorable safety profile in our study, including patient selection, routine clinical monitoring, and use of Lactobacillus and Bifidobacterium species with established safety records. Nevertheless, the absence of microbiome sequencing and strain-level surveillance limited our ability to evaluate colonization dynamics, probiotic translocation risk, or changes in SCFA-producing taxa.

The modest increase in diarrhea frequency in the combination group is clinically important given the laxative properties of the Tongli Gongxia formula. Most episodes were manageable through dose adjustment and supportive care, and severe diarrhea requiring treatment discontinuation occurred in 3% of combination-treated patients. Although improved intestinal transit may be part of the intended therapeutic strategy in TCM practice, excessive diarrhea can aggravate fluid loss, electrolyte disturbance, and hemodynamic instability in vulnerable SAP patients. Therefore, diarrhea should be regarded as a safety outcome requiring proactive monitoring rather than only as a benign treatment effect (Li et al., 2016).

Our study has several strengths compared to much of the existing literature on complementary approaches to SAP management. The use of propensity score matching reduced confounding by indication, a major threat to validity in observational studies of treatment associations. Attention to temporal factors, including treatment initiation timing and concurrent therapies, allowed for more nuanced adjustment than simple cross-sectional comparisons. The comprehensive measurement of barrier biomarkers and day 7 SCFA metabolites provided clinically relevant biological endpoints beyond symptoms or global outcomes alone. The relatively large sample size for a single-center study of a specialized intervention provided adequate statistical power for the primary outcomes and enabled meaningful multivariable adjustment. Finally, detailed documentation of concurrent therapies and sensitivity analyses enhanced interpretability of findings (Rosenbaum, 2002; Austin, 2011).

This study has several important limitations. The retrospective observational design limits causal inference despite statistical adjustments, and unmeasured confounders may have introduced residual bias. The single-center setting constrains generalizability. The exposure was heterogeneous because probiotic products, viable count stability, delivery route, herbal dose ranges, syndrome-differentiated modifications, and oral, nasojejunal, or rectal routes reflected real-world clinical practice rather than a uniform trial protocol. Although **Supplementary Table 1** summarizes the main intervention characteristics, complete patient-level daily TCM modification records were not consistently retrievable from legacy unstructured electronic medical records. Therefore, residual exposure heterogeneity remains an important limitation and may affect reproducibility and external validity. The absence of separate probiotic-only and Tongli-only comparator groups prevents determination of whether observed associations were attributable to probiotics, Tongli Gongxia therapy, treatment timing, or the combination as a whole, and synergy cannot be inferred. Baseline fecal SCFA samples were unavailable, so SCFA results represent day 7 between-group differences rather than treatment-induced metabolic restoration. The lack of metagenomic or 16S rRNA sequencing data limits mechanistic interpretation regarding microbial rebalancing or enrichment of SCFA-producing bacteria. Barrier biomarkers provide only indirect evidence of intestinal function, and the study was underpowered for several secondary clinical outcomes. Despite these limitations, our findings suggest that early receipt of probiotics combined with Tongli Gongxia Chinese medicine alongside standard SAP care may be associated with intestinal barrier biomarker improvement and higher day 7 SCFA concentrations. Rigorous validation through well-designed prospective randomized controlled trials is essential to establish causality, optimize patient selection, standardize intervention protocols, and guide clinical implementation.

In conclusion, this retrospective cohort study provides hypothesis-generating evidence that probiotics combined with Tongli Gongxia Chinese medicine were associated with greater reductions in intestinal barrier dysfunction biomarkers, higher day 7 fecal SCFA concentrations, and shorter ICU length of stay in SAP patients. Because of the observational design, heterogeneous exposure, absence of baseline SCFA measurements, and lack of microbiome sequencing, the findings should be interpreted cautiously and require confirmation in prospective randomized controlled trials before routine clinical implementation.

## Data Availability

The original contributions presented in the study are included in the article/**Supplementary Material**. Further inquiries can be directed to the corresponding author.
