# Supplementary material for: Associations of probiotics combined with Tongli Gongxia Chinese medicine with intestinal barrier biomarkers and day 7 short-chain fatty acids in patients with severe acute pancreatitis
Source: Front Cell Infect Microbiol. 2026 Jun 29;16:1837621. doi: 10.3389/fcimb.2026.1837621 (PMC13357428; doi:10.3389/fcimb.2026.1837621)
Supplement: Supplementary file 1 [file Table1.docx]

**Supplementary Table S1**

**Summary of intervention characteristics and exposure heterogeneity in the combination therapy group (n=100)**

| **Intervention category** | **Specific components / parameters** | **Dose range / specifications** | **Frequency / distribution, n (%)** |
| --- | --- | --- | --- |
| **1. Probiotic therapy** | Documented strains: Lactobacillus acidophilus and Lactobacillus rhamnosus | Present in the multi-strain formula | 100 (100%) |
|  | Documented strains: Bifidobacterium animalis and Bifidobacterium bifidum | Present in the multi-strain formula | 100 (100%) |
|  | Total daily dose | 2-3 x 10^10 CFU | 100 (100%) |
|  | Administration route: nasojejunal tube | - | 68 (68%) |
|  | Administration route: oral | - | 32 (32%) |
| **2. Tongli Gongxia TCM therapy** | Core formula component: Rheum palmatum / Rhei Radix et Rhizoma (Rhubarb / 大黄) | 9-15 g | 100 (100%) |
|  | Core formula component: Magnolia officinalis (Magnolia bark / 厚朴) | 9-12 g | 100 (100%) |
|  | Core formula component: Citrus aurantium (Bitter orange / 枳实) | 9-12 g | 100 (100%) |
|  | Core formula component: Natrii Sulfas / Mirabilite (芒硝)* | 6-12 g | 85 (85%) |
|  | Syndrome-based addition: Scutellaria baicalensis (黄芩) and/or Coptis chinensis (黄连) | 6-10 g; heat-clearing modification | 42 (42%) |
|  | Syndrome-based addition: Radix Paeoniae Alba (白芍) | 10-15 g; cramp-relieving modification | 35 (35%) |
|  | Administration route: nasojejunal tube, granule concentrates | - | 65 (65%) |
|  | Administration route: oral, traditional decoction or granules | - | 25 (25%) |
|  | Administration route: rectal enema | - | 10 (10%) |

**Note:** ** Natrii Sulfas / Mirabilite was withheld in selected patients based on syndrome differentiation, mainly in cases with excessive baseline diarrhea or relevant contraindications. Syndrome-based modifications were individualized in routine clinical practice. Therefore, only the most frequently documented additions are summarized here to characterize the major sources of exposure heterogeneity.*
